# Stem Cells Grown in Osteogenic Medium on PLGA, PLGA/HA, and Titanium Scaffolds for Surgical Applications

**DOI:** 10.1155/2010/831031

**Published:** 2010-12-23

**Authors:** Annalia Asti, Giulia Gastaldi, Rossella Dorati, Enrica Saino, Bice Conti, Livia Visai, Francesco Benazzo

**Affiliations:** ^1^IRCCS Foundation, Orthopaedic and Traumatology Department, SMEC Department, San Matteo Hospital Institute, University of Pavia, 27100 Pavia, Italy; ^2^Center for Tissue Engineering (CIT), University of Pavia, Via Ferrata 1, 27100 Pavia, Italy; ^3^Department of Physiology, Section of Human Physiology, University of Pavia, Via Forlanini 6, 27100 Pavia, Italy; ^4^Department of Pharmaceutical Chemistry, University of Pavia, Viale Taramelli, 27100 Pavia, Italy; ^5^Department of Biochemistry, University of Pavia, Viale Taramelli 3/b, 27100 Pavia, Italy

## Abstract

Pluripotent adipose tissue-derived stem cells (hASCs) can differentiate into various mesodermal cell types such as osteoblasts, chondroblasts, and myoblasts. We isolated hASCs from subcutaneous adipose tissue during orthopaedic surgery and induced the osteogenic differentiation for 28 days on three different synthetic scaffolds such as polylactide-co-glycolide (PLGA), polylactide-co-glycolide/hydroxyapatite (PLGA/HA), and trabecular titanium scaffolds (Ti6Al4V). Pore size can influence certain criteria such as cell attachment, infiltration, and vascularization. The aim of this study was to investigate the performance of PLGA and PLGA/HA scaffolds with a higher porosity, ranging between 75% and 84%, with respect to Ti scaffolds but with smaller pore size, seeded with hASCs to develop a model that could be used in the treatment of bone defects and fractures. Osteogenesis was assessed by ELISA quantitation of extracellular matrix protein expression, von Kossa staining, X-ray microanalysis, and scanning electron microscopy. The higher amount of protein matrix on the Ti scaffold with respect to PLGA and PLGA/HA leads to the conclusion that not only the type of material but the structure significantly affects cell proliferation.

## 1. Introduction

Stem cells have become the main cell source for tissue repair because they meet several major cell therapy requirements that differentiated primary cells do not meet. They are defined by their self-renewal, differentiation capacity, and they are able to proliferate in culture without losing their potential to form tissue [1]. The use of stem cells in regenerative medicine has received a great deal of interest in recent years [2], and a promising approach is to promote tissue regeneration by transplanting tissue engineered constructs made of a biofactor (cell/gene and/or proteins) grown on a porous structure known as scaffold [3]. The scaffold provides mechanical support and serves as a substrate upon which cells proliferate and undergo differentiation. Synthetic scaffolding made of several different promiscuous materials such as titanium, hydroxyapatite, and polymers, has been used in the treatment of bone defects and fractures for over 100 years [4]. Partially resorbable polymers such as poly-alfa-hydroxy acids are now being introduced, which allow for new bone growth; these novel polymers have been shaped into self-reinforcing screws, dowels, rods, and spacers and have been used with some success in large-bone fracture fixations. These materials have quite good mechanical characteristics in terms of stiffness and compression resistance. Investigations into synthetic and natural inorganic ceramic materials such as HA as coated scaffold material have been employed mostly in bone tissue engineering [5]. This is because these ceramics resemble the natural inorganic component of bone and have osteoconductive properties [6]. Recent developments have led to the interest in the potential of porous HA as synthetic bone graft [7]. HA exhibits a strong propensity for attracting osteoblasts but possesses a low resorption rate *in vivo* and is brittle, especially in highly porous forms. In order to alleviate some of these inherent issues, while still maintaining its benefits, HA has been combined with several natural and synthetic polymers such as PLGA to produce composite scaffolds. The addition of biodegradable PLGA to HA would allow for better manipulation, biocompatibility, and control over both the macro- and microstructure in shaping composites to fit bone defects. In addition, PLGA could be used as a binder for HA to reduce brittleness of ceramics. PLGA/HA composites are promising materials for bone grafts and have been extensively investigated [8].

Polymeric 3D scaffolds have several advantages since they permit a precise selection of the material and consequently of the nanostructure and the architecture of scaffolds. Moreover, they have been widely studied in the last years, mainly due to their good biocompatibility (their use also had its origin and has been consolidated in the pharmaceutical field), their chemical versatility, and good biological performances; they also do not imply danger of immunogenic reactions or possibility of disease transmission. They biodegrade by random chain scission generating monomers of lactic and glycolic acid that are eliminated through the metabolic pathways. The intrinsic properties of the raw material play a strategic role in the production, structure, and morphology and, consequently, in the functional performances of the polymer scaffold [9]. To act as an artificial ECM, the structure and the surface morphology of the scaffolds have to meet general requirements specific for the targeted tissue: (i) interconnected pores to ensure cells growth and nutrients and metabolic waste transport flow; (ii) three-dimensional architecture; (iii) suitable mechanical properties; (iv) suitable surface chemistry; (v) controllable biodegradation and bioresorbability [10]. The scaffold shape should also facilitate cell seeding and attachment and promote cell proliferation and differentiation [11]. Moreover, the bioresorbable scaffold should present mechanical properties (strength and stiffness) equivalent to those of the host tissue until the bioresorbable scaffold matrix is substituted by the new tissue [12].

Trabecular titanium is an inert non-biodegradable material with an excellent biocompatibility [2]; it has been utilized predominantly for long bone defects because of its excellent compressive strength. Not only does the scaffold shape provide a substrate on which bone can grow, but also scaffold geometry influences critical environmental properties such as the feasibility of vascular ingrowth and resistance to fibrous tissue infiltration [4]. Porosity is a measure of the open pore volume within the matrix, often called the void fraction. Open pores have cellular access on both sides and allow for liquid flow and transport of nutrients through the porous matrix [13].

Pore size is referred to the distance between solid sections of the porous matrix; it is typically reported as the diameter of circular pores or the major axis for noncircular pores. Pore size affects cell binding, migration depth of cellular in-growth, cell morphology, and phenotypic expression [14]. Scaffolds with mean pore size ranging from 20 *μ*m to 1500 *μ*m have been used in bone tissue engineering applications [15]. By facilitating capillary formation, pores greater than 300 *μ*m lead to direct osteogenesis, while pores smaller than 300 *μ*m can encourage osteochondral ossification [15, 16]. 

Pore size not only affects cell growth but also affects scaffold properties; for example, the elasticity of microporous scaffold increases as the number of pores within the scaffold increases [13, 17]. The pore architecture of polymer scaffold resulted to be between 300 and 350 *μ*m, porosity 65–70%; the average diameter of the cell pores used in Ti6Al4V construct is 640 *μ*m, and the structure has an average porosity of 65%.

Bone tissue engineering techniques based on autogenous cell/tissue transplantation would eliminate problems of donor scarcity, supply limitation, pathogen transfer, and immune infection [18].

The aim of this study was to evaluate the adhesion and osteogenic differentiation of hASCs grown on polylactide-co-glycolide (PLGA), polylactide-co-glycolide/hydroxyapatite (PLGA/HA), and on trabecular titanium scaffolds (Ti6Al4V) by comparing the analysis of indicators of osteoblastic phenotype such as cell adhesion on different scaffolds, the extraction and measurement of type-I collagen (COL I) and alkaline phosphatase (ALP). Scanning Electron Microscopy (SEM) of all types of scaffolds before and after colonization with cells, von Kossa staining, and X-ray microanalysis were performed to detect the calcified extracellular matrix production.

## 2. Materials and Methods

### 2.1. Materials

LGA polymer (PLGA 8515 DLG 7E, Mw 120 kDa, Mn 97 kDa) was purchased from Lakeshore Biomaterials, Birmingham (USA). Salt (NaCl, Mw 58.443 g/mol, solubility in water 36 g/100 mL at 20°C) and 1,4-Dioxane, used for the preparation of PLGA scaffolds, were obtained from Carlo Erba, Milan (Italy). Hydroxyapatite (HA) nanopowder <200 nm was purchased from Sigma-Aldrich. The water used in the preparation of scaffold was distilled and filtered through 0.22 *μ*m Millipore membrane filters (Millipore Corporation, Massachusett, USA). Unless specified, all other solvents and reagents were of analytical grade.

### 2.2. Preparation and Characterization of PLGA, PLGA/HA Scaffolds

**Figure 1:**
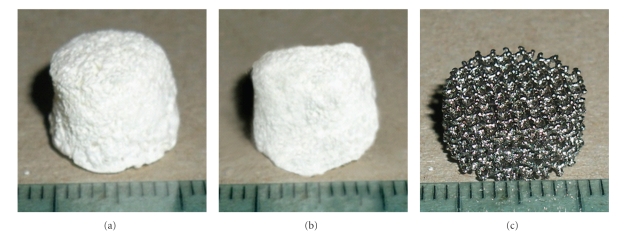
Images of PLGA (a) and PLGA/HA (b) scaffolds obtained using the porogen particle leaching method as reported in “Materials and methods”; (c) image of trabecular titanium scaffold (Ti6Al4V).

PLGA and PLGA/HA scaffolds were prepared by solvent/casting particulate leaching method as explained in a previous work [19]. Briefly, the scaffold preparation was performed as follows: 700 *μ*L of PLGA solution (15% w/v in 1,4-Dioxane) or of a suspension of 5% HA in the PLGA were cast drop by drop into Teflon moulds (cylindrical vials with a diameter of 10 mm) filled with 700 mg of NaCl porogen particles with 600–1180 *μ*m diameter. The mould containing the porogen and the polymer solution was first maintained at room temperature (RT) overnight to permit the diffusion of the polymer solution through the porogen particles, and then it was placed at −25°C for 24 hours. The frozen porogen/polymer mixture was freeze-dried at −50°C for 12 hours to completely remove the solvent. The scaffolds were dialyzed in water (200 mL) at RT for 21 days to remove the porogen particles. The water was changed three times a day for the first week and then once a week. After dialysis the scaffolds were freeze-dried at −50°C overnight. The prepared scaffolds were stored in a dessicator at −25°C. The scaffolds have a height of 6 mm and a diameter of 12 mm (Figures 1(a) and 1(b)). The same preparation process has been used to obtain strip samples of 5 × 5 × 15 mm that underwent tension tests.

### 2.3. Characterization of PLGA, PLGA/HA Scaffolds

#### 2.3.1. Density and Porosity Determination

The density and porosity values of the PLGA and PLGA/HA polymer scaffolds were measured using a modified liquid displacement method [20] with ethanol as the displacement liquid.

A weighted polymer scaffold (W) was immersed in a graduated cylinder containing a known volume (V_1_) of ethanol. The sample was kept in the nonsolvent for 10 min, and then a set of evacuation-repressurization cycles was conducted to force the ethanol into the pore structure.

Cycling was continued until no air bubbles were observed leaving the scaffold surface. The total volume of the ethanol and ethanol-soaked scaffold was then recorded as V_2_. The volume difference, (V_2_−V_1_), represented the volume of the scaffold skeleton. The ethanol-soaked scaffold was then removed from the cylinder and the residual ethanol volume was recorded as V_3_. The volume (V_1_−V_3_), that is, the ethanol volume retained in the porous scaffold, was defined as the pore volume of the scaffold. The total volume of the scaffold was calculated as follows:

(1) $$\text{V}\;=\;\left({\text{V}}_{2}-{\text{V}}_{1}\right)+\;\left({\text{V}}_{1}-{\text{V}}_{3}\right)=\;{\text{V}}_{2}-{\text{V}}_{3}.$$

The density of the scaffold (*d*) was expressed as

(2) $$d\;=\;\text{W}/\left({\text{V}}_{2}-{\text{V}}_{3}\right).$$

And the porosity of the scaffold (*ε*) expressed as percentage (%) was calculated by

(3) $$\varepsilon \;\left(\text{\%}\right)\;=\;\left({\text{V}}_{1}-{\text{V}}_{3}\right)/\left({\text{V}}_{2}-{\text{V}}_{3}\right)\;*100.$$

The density and porosity determined in triplicate (*n* = 3) and expressed as mean  ± standard deviation are reported in Table 1.

#### 2.3.2. Mechanical Tests

Cylindrical samples (*∼*6 mm diameter × 12 mm height) underwent compression tests whereas strip samples (~5 × 5 × 15 mm) underwent tensile tests.

 All the mechanical tests were performed using an electromagnetic testing machine (Enduratec ELF3200, Enduratec-Bose, Minnetonka, MN, USA), equipped with a load cell of 220N for evaluation of compression and tensile resistance, under displacement control, at a velocity of 0.1 mm/s. Different grips were used with the machine depending on the test configuration, that is, compression or tension. Compression test has been carried out by application on the cylindrical scaffolds, of same loadings with opposite directions both directed towards the interior part of the scaffold. This allows the uniform distribution of forces, on an orthogonal plan, inside the scaffold structure. In the presence of an elastic material, this determines shortening of the matrix in its axial direction and widening of the matrix in its radial direction. 

The results of compression tests and tensile tests are reported in Table 2 as elastic modulus (E_c1_, E_c2_, E_c3_, and E_t_) extracted from the linear regions of the stress strain curves.

### 2.4. Titanium Scaffolds

Trabecular Titanium scaffolds (Ti6Al4V) were provided by manufacturer Lima-Lto S.p.A. (Lima, Villanova di San Daniele del Friuli, Italy). An innovative multiplanar hexagonal cell structure imitating the cell structure of the trabecular bone was developed, and its morphology and dimension have been optimized to improve vascularization and therefore maximize osteointegration. 

Previous studies have shown that the minimum pore size to improve osteointegration is 300 *μ*m. Moreover, cells grew at a significantly faster rate into drill channels having a diameter of 600 *μ*m than channels of diameters of 300, 400, 500, and 1000 *μ*m [21]. The average diameter of the cell pores used in Ti6Al4V construct is 640 *μ*m; the structure has an average porosity of 65% [22]. The Trabecular Titanium scaffolds (Ti) used have a height of 6 mm and a diameter of 12 mm (Figure 1(c) ); the value of elastic modulus of trabecular Titanium has been reported by Marin et al. [22].

### 2.5. Human Adipose Derived Stem Cells (hASCs)

Stem cells were prepared from subcutaneous adipose tissue obtained from healthy donors during orthopaedic surgery. Informed consent was obtained before surgical intervention. Briefly, the tissue was finely minced and then incubated in digestion buffer (0.01% collagenase type II in DMEM F12-HAM medium supplemented with 10% FBS, 100 U penicillin/streptomycin, amphotericin) for 1 h at 37°C while vigorously shaking. At the end of the incubation time, five volumes of DMEM F12-HAM were added to neutralize collagenase, and the suspension was centrifuged at 1200 rpm for 10 min. The resulting pellet, containing hASCs, was suspended in DMEM F12-HAM supplemented with 10% FBS, 100 U penicillin/streptomycin, and amphotericin (control medium, CM). The hASCs were initially cultured in CM up to 95% confluence in a humidified atmosphere of 95% air with 5% CO2 at 37°C. The adherent cells were trypsinized, and 1 × 10^5^ hASCs per 100 mm^2^ were seeded in flasks. These passages were repeated three times.

### 2.6. Flow Cytometric Surface Markers Analysis

hASCs were phenotypically characterized by flow-cytometry; fluorescein isothiocyanate (FITC) or phycoerythrin (PE)-conjugated monoclonal antibodies, specific for CD45, CD34, CD90 (BD PharMingen, San Diego, Calif, USA), CD73, CD105 (Serotec, Kidlington, Oxford, UK), were used.

Appropriate, isotype-matched, nonreactive fluorochrome-conjugated antibodies were employed as controls. An analysis of cell populations was performed by means of direct immunofluorescence with an FACScalibur flow cytometer (BD PharMingen), and data was calculated using CellQuest software (BD Pharmingen).

### 2.7. Cell Seeding and Culture Conditions

At confluence, the cells were trypsinized and inoculated onto each scaffold as follows: a drop of 50 *μ*L containing 5 × 10^5^ cells was placed on the top of the scaffolds which were placed in 24 wells (Costar, Corning Inc., NY, USA) and allowed to be adsorbed by the porous substrates for 2 hours before the CM was added. For osteogenic differentiation, 5 cell/scaffold constructs for each type of scaffold were switched over to osteogenic medium (OM) (DMEM F12-HAM containing 15% FBS, 10 mM beta-glycerophosphate, 100 nM dexamethasone, 0.05 mM ascorbic acid, antibiotics, and amphotericin). The scaffolds were maintained in OM for 28 days.

3-(4,5-dimethylthiazole-2-yl)-2,5-diphenyltetrazolium bromide test.

 To evaluate the mitochondrial activity of the seeded cells, that is, the cell viability on the PLGA, PLGA/HA, and 3D Ti scaffolds during the culture period, a test with 3-(4,5-dimethylthiazole-2-yl)-2,5-diphenyl tetrazolium bromide (MTT) (Sigma-Aldrich) was performed on days 1, 3, 14, and 28 (end of the culture period). The culture medium was replaced with a 0.5 mg/mL solution of MTT in phosphate-buffered saline (PBS) (137 mM NaCl, 2.7 mM KCl, 4.3 mM Na_2_HPO_4_, 1.4 mM KH_2_PO_4_, pH 7.4), and the cell cultures were incubated for 4 hs. Viable cells are able to reduce MTT into formazan crystals. After removing the MTT solution, to solubilize the formazan products, 500 *μ*L of dimethyl sulfoxide (Sigma-Aldrich) were added, and the well plate containing the cultured 3D scaffolds was agitated for 20 min on a shaker. Aliquots of 200 *μ*L were sampled, and the related absorbance values were measured at 570 nm with a microplate reader (BioRad Laboratories, Hercules, Calif, USA). A standard curve of cell viability was used to express the results as percentage.

### 2.8. DNA Content

At the end of incubation (28 days), cells present on the scaffolds were lysed by a freeze-thaw method in sterile deionized distilled water. The released DNA content was evaluated with a fluorometric DNA quantification kit (PicoGreen, Molecular Probes, Eugene, Ore, USA). A DNA standard curve, obtained from a known amount of osteoblasts, was used to express the results as cell number per scaffold.

### 2.9. Scanning Electron Microscopy (SEM)

SEM was performed on PLGA, PLGA/HA and on trabecular titanium scaffolds before and after 28 days of incubation with hASCs cells. The scaffolds were fixed in glutaraldehyde 2.5% and Na-cacodylate buffer at pH 7.4 for about 2 hrs and then washed with Na-cacodylate buffer for 30 minutes. The dehydration process was performed using an increasing ethanol concentration (from 50% to 100%). Samples were then submitted to critical point drying with CO_2_, mounted on aluminium stubs, and gold sputtered (degree of purity 99.9%) under argon atmosphere to allow adequate gold coating of the internal surface of porous structure (Sputter coater BALZER). Observations and micrographs were performed with an SEM Cambridge Stereoscan, operating at 20 kV. 

#### 2.9.1. Set of Purified Proteins and Antibodies for ELISA Assay

Type-I collagen was purified as described previously [23, 24]; osteocalcin was acquired from Biomedical Technologies (Stoughton, Mass, USA), and alkaline phosphatase was purchased from Sigma-Aldrich, Inc. Dr. Larry W. Fisher (http://csdb.nidcr.nih.gov/csdb/antisera.htm, National Institutes of Health, Bethesda, Md, USA) provided us with the rabbit polyclonal antitype-I collagen, anti-osteocalcin, and antialkaline phosphatase.

#### 2.9.2. Extraction of the Extracellular Matrix Proteins from the Cultured Scaffolds and ELISA Assay

On days 3 and 28, in order to evaluate the amount of the extracellular matrix constituents through the scaffolds surface, the scaffolds were washed extensively with sterile PBS in order to remove the culture medium and then incubated for 24 hs at 37°C with 1 mL of sterile sample buffer (20 mM Tris-HCl, 4 M GuHCl, 10 mM EDTA, 0,066% [w/v] SDS, pH 8.0). At the end of the incubation period, the sample buffer aliquots were removed, and then the 3D Ti, PLGA, and PLGA/HA scaffolds were centrifuged at 4000 rpm for 15 min in order to collect the sample buffer entrapped inside the pores. The total protein concentration in the culture system was evaluated by the BCA Protein Assay Kit (Pierce Biotechnology, Inc., Rockford, Ill, USA). The total protein concentration was 0,573 ± 0,021 mg/mL on Ti scaffolds, 0, 120 ± 0,010 mg/mL on PLGA scaffolds, and 0, 414 ± 0,018 mg/mL on PLGA/HA scaffolds. After matrix extraction, the scaffolds were incubated, once again, for 24 hs at 37°C with 1 mL of sterile sample buffer, and no protein content was detected. Calibration curves to measure type-I collagen and alkaline phosphatase were performed. Microtiter wells were coated with increasing concentrations of each purified protein, from 10 ng to 2 *μ*g, in coating buffer (50 mM Na2CO3, pH = 9.5) overnight at 4°C. Some of the wells were coated with bovine serum albumin (BSA) as a negative control. In order to measure the extracellular matrix amount of each protein by an ELISA assay, microtiter wells were coated, overnight at 4°C, with 100 *μ*L of the previously extracted extracellular matrix (20 *μ*g/mL in coating buffer). After three washes with PBST (PBS containing 0.1% [v/v] Tween 20), the wells were blocked by incubating with 200 *μ*L of PBS containing 2% (w/v) BSA for 2 hs at 22°C. The wells were subsequently incubated for 1.5 hs at 22°C with 100 *μ*L of the L. Fisher's anti-type-I collagen and anti-alkaline phosphatase rabbit polyclonal antisera (1 : 500 dilution in 1% BSA). After washing, the wells were incubated for 1 h at 22°C with 100 *μ*L of HRP-conjugated goat antirabbit IgG (1 : 1000 dilution in 1% BSA). The wells were finally incubated with 100 *μ*L of substrate solution (phosphate-citrate buffer with *o*-phenylenediamine dihydrochloride). The colour reaction was stopped with 100 *μ*L of 0.5 M H_2_SO_4_, and the absorbance values were measured at 490 nm with a microplate reader (BioRad Laboratories). An underestimation of absolute protein deposition is possible because the sample buffer, used for matrix extraction, contained sodium dodecyl sulphate, which may have interfered with protein adsorption during ELISA assay. The amount of extracellular matrix constituents throughout the scaffolds was expressed as pg/(cell × scaffold).

#### 2.9.3. Evaluation of Calcium Deposition

Monolayers of cells grown on culture plates were rinsed with PBS and fixed in 4% paraformaldehyde for 1 h at room temperature. The cells were then incubated in 5% silver nitrate for 30 minutes in the dark, rinsed with distilled water, and exposed to ultraviolet light for 1 h. Secreted calcified extracellular matrix was observed as black nodules.

X-ray microanalysis of the samples was run to detect the presence of Ca, P and their location within the scaffolds. The images were obtained with a Cambridge Stereoscan 250, Scanning Electron Microscope.

### 2.10. Statistical Analysis

Data are presented as mean ± SD. Statistical analyses were performed using the one-way ANOVA method followed by Newman-Keuls'Q test (Graph-Pad Prism 4.0).

## 3. Results

### 3.1. PLGA, PLGA/HA Scaffolds Characterization

Table 1 reports the porosity and apparent density of the polymeric scaffolds. The PLGA scaffolds resulted to have significantly higher porosity than PLGA/HA scaffolds. This is probably due to the presence of HA that is mixed as nanosize powder to the polymeric solution during the scaffold preparation process. The pore architecture of polymer scaffold examined by scanning electron microscopy shows the presence of interconnected pores whose diameter is between 200 and 350 *μ*m. As shown in Table 1, the presence of HA reduces both porosity and pore size of scaffolds.

As long as mechanical properties are concerned, compression up to 50% of the initial length resulted in plastic deformation without failure. Conversely, tension tests resulted in specimen failure although the strain level for failure was variable depending on the scaffold. 

Compression stress-strain curves of PLGA scaffolds showed three linear regions. Compression stress-strain curves of PLGA+HA showed two linear regions. All tension tests showed one linear region before yield and failure. Compression and tension moduli as derived from all the linear regions are reported in Table 2. The presence of HA in the composite scaffolds does not seem to improve the compression properties of polymeric scaffolds, while tensile properties are improved by addition of HA to the polymeric structure.

### 3.2. Flow Cytometry

**Figure 2:**
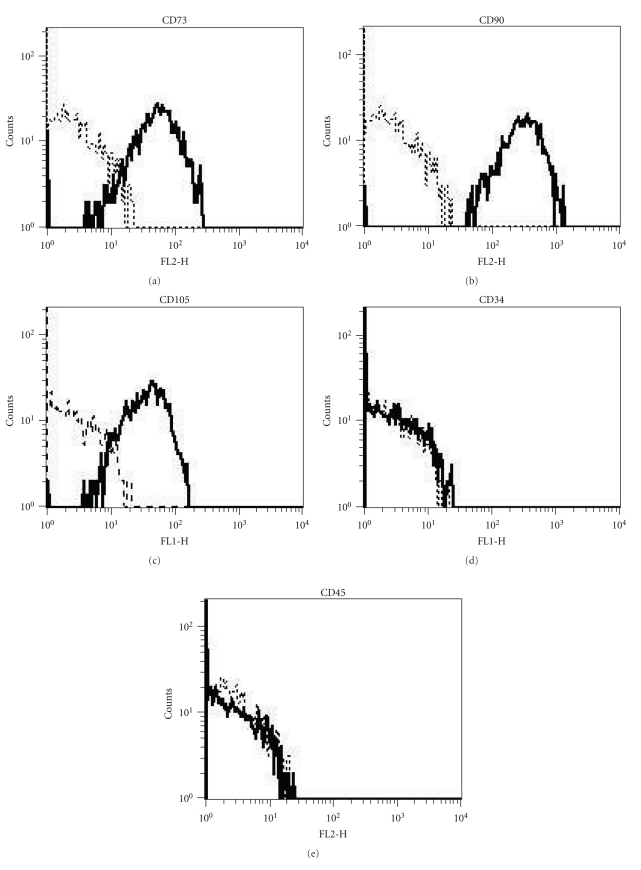
Phenotypic characterization of hASCs: CD105, 73, and 90 mesenchymal stem cells markers are positive; CD34 and 45 haematopoietic stem cells markers are negative.

The surface phenotype of hASCs was analyzed by flow cytometry at passage 3 (P3), and resulted to be in agreement with previous reports [25, 26]. In particular, by the third passage, contamination with hematopoietic cells was no longer detectable, and more than 98% of the cells expressed the MSC typical surface marker pattern. In detail, hASCs were positive for CD73, CD90, and CD105 surface antigens and negative for CD34 and CD45 molecules [27, 28] (Figure 2).

### 3.3. Monolayer Culture of hASCs

**Figure 3:**
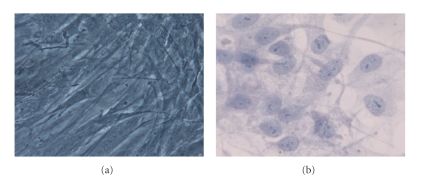
(a) hASCs after the 3rd passage in control medium show a fibroblast-like shape; (b) hASCs in osteogenic medium after 28 days show a more spherical shape if compared to the undifferentiated cells (Toluidine blue). Mag. 10x.

In a monolayer culture prior to osteogenic induction, hASCs showed an elongated, fibroblastic appearance (Figure 3(a)). After 28 days of cell culture in OM, the hASC morphology differentiated to osteoblasts, changing into a rounder, cuboidal shape (Figure 3(b)). Furthermore, mineralization was determined qualitatively for calcium deposition by von Kossa staining (Figure 7(a)); positive staining was detected by the appearance of black nodules (Figure 7(b)).

### 3.4. Cell Morphology

**Figure 4:**
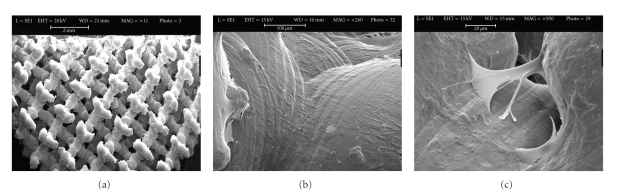
Scanning electron microscopic images of unseeded titanium scaffolds (a) and seeded titanium scaffolds (Ti6Al4V) with hASCs in osteogenic medium (b). Panel A shows the innovative multi-planar hexagonal structure of the scaffold imitating the structure of the trabecular bone, bar = 2 mm. In Panel B, cells appear to cover the surface of the trabecular scaffold uniformly and completely, bar = 100 *μ*m; (c) Extracellular matrix between pores, bar = 20 *μ*m.

**Figure 5:**
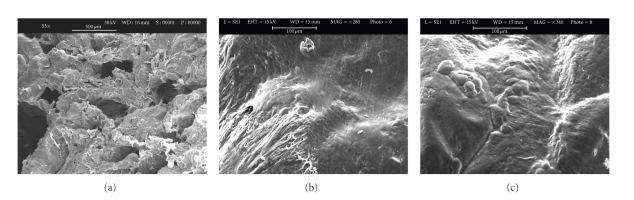
Scanning electron microscopic images of unseeded PLGA scaffold (a), bar = 500 *μ*m and seeded PLGA scaffold (b and c) with hASCs in osteogenic medium for 28 days. Panel b shows cells embedded in their extracellular matrix over the scaffold surface bar = 100 *μ*m. Panel c shows round cells bar = 100 *μ*m.

**Figure 6:**
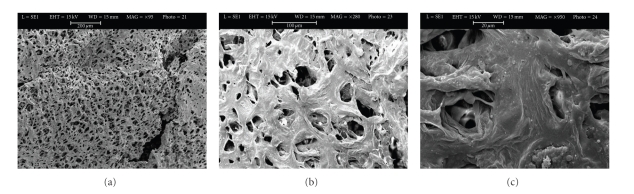
Scanning electron microscopic images of unseeded PLGA/HA scaffold (a), bar = 200 *μ*m and seeded PLGA/HA scaffolds (b and c) with hASCs in osteogenic medium for 28 days. In panel B, cells appear have a round morphology, bar = 100 *μ*m. Panel C, at greater magnification, clusters of cells embedded in their matrix and inside the pores of the scaffold, bar = 20 *μ*m.

Cells cultured on the 3D Ti, PLGA, and PLGA/HA scaffolds were observed by SEM (Figures 4, 5 and 6). Figures 4, 5, and 6 are each a representative image of 28 days of cell culture in OM showing adherence of cells to the surface on the 3 types of scaffolds. In particular, the cells homogeneously covered the surface and spanned to the neighbouring fibers on the 3D Ti scaffold (Figure 4(b)) and PLGA scaffolds (Figure 5(b)). At higher magnification on the Ti scaffold (Figure 4(c)) cells are embedded within a dense layer of ECM that also forms a bridge between the porous structures.

On PLGA sample, cellular processes covered almost the entire scaffold surface in an abundant ECM (Figure 5(c)). On the PLGA/HA, some cells were present on the surface (Figure 6(b)), but the majority of cells were completely embedded in ECM, filling the porous structure, as can be observed at higher magnification (Figure 6(c)).

To evaluate the cell viability on the PLGA, PLGA/HA, and Ti scaffolds during the culture period, an MTT test was performed. On days 1, 3, 14, and at the end of the culture period, the average cell viability was in the 86–91% range with no statistically significant difference in the cell viability (*P* > .05) among all types of scaffold at each culture period.

### 3.5. Cell Attachment

To assess whether the different types of scaffolds could influence the initial cell attachment and thus the ECM deposition, the number of osteoblasts attached to every type of scaffold was detected earlier on days 1 and 3 and later on day 28. The longer incubation time was chosen to allow the *in vitro* cell production of detectable bone proteins. The percentage of cell attachment was about 20% ± 2.5% (on day 1) and 35% ± 2.2% (on day 3) for all types of scaffolds, showing no significant difference (*P* > .05). After 28 days of cell culture, a significantly consistent increase in the measurement of DNA content was detected on the titanium 3D scaffold when compared to PLGA and PLGA/HA scaffolds. On the Ti, the cell number per scaffold rose to 4,5 × 10^5^ ± 0,4 × 10^2^, whereas on PLGA scaffolds it reached 3,8 × 10^5^ ± 0,2 × 10^2^ (*P* < .05). The number of cells attached to PLGA/HA was around 3,7 × 10^5^ ± 0,15 × 10^2^.

### 3.6. Characterization of the Calcified ECM Deposition

To evaluate the amount of the ECM constituents produced throughout PLGA, PLGA/HA, and Ti scaffolds, an ECM extraction was performed on days 3 and 28 of incubation. Unfortunately, on day 3 even if the total protein content was determined, the levels of the specific bone proteins were too low to be detected in all types of scaffolds. At the end of the culture period the deposition of ALP, type-I collagen, and osteocalcin throughout the Ti scaffolds was significantly higher (*P* < .05) in comparison with the culture grown on the PLGA and PLGA/HA scaffolds (Table 3). The enhancement of protein deposition was particularly marked for type I collagen, which was fivefold and threefold greater when compared with the PLGA and PLGA/HA samples, respectively (Table 3). The level of the ALP deposition was almost twofold higher on the Ti scaffolds with respect to PLGA and PLGA/HA; the deposition of osteocalcin, which is known to be a mineralization marker, was significantly lower on PLGA and PLGA/HA samples when compared to Ti scaffolds.

**Figure 7:**
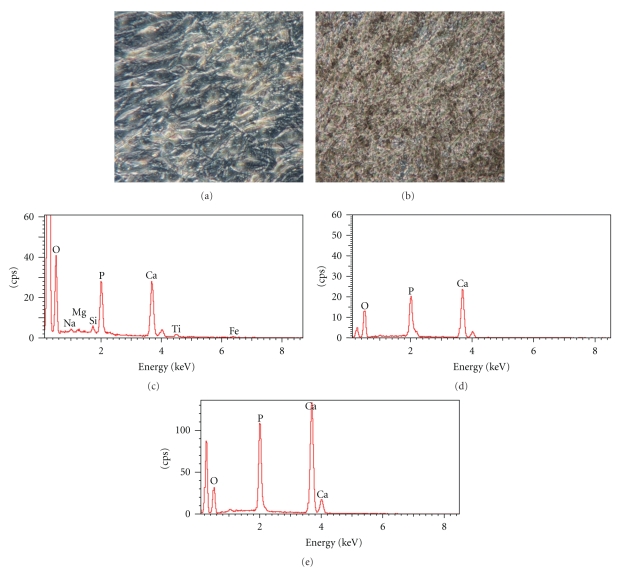
von Kossa staining of hASCs grown in osteogenic medium for 28 days in a culture monolayer. (a) Negative control, (b) positive sample; the secreted calcified extracellular matrix are shown as black nodules Mag. 20x. (c) X-ray microanalysis performed on trabecular titanium, (d) PLGA scaffolds, (e) PLGA/HA scaffolds seeded with hASCs in osteogenic medium for 28 days. Calcium and Phosphatum peaks were detected, inferring that hydroxyapatite was formed.

The qualitative evaluation of the calcium deposition was performed by X-ray microanalysis on cells grown on PLGA, PLGA/HA, and Titanium scaffold (Figures 7(c), 7(d), 7(e)) that revealed an increased presence of calcium and phosphorus inferring that calcium phosphate (hydroxyapatite) had been deposited. No significant differences among the scaffolds were observed.

## 4. Discussion

In this paper, we studied the adhesion and the differentiation of hASCs grown in osteogenic medium for 28 days on PLGA, PLGA/HA, and Trabecular Titanium scaffolds. The biocompatibility of the biomaterial is very closely related to the cell behaviour in contact with the biomaterial and particularly to cell adhesion on the biomaterial surface [3, 29]. The material surface can influence cell reaction through changes in the cytoskeleton, a network of protein filaments extending through the cell cytoplasm within eukaryotic cells [29]. It is known that cell behaviour and interaction with a biomaterial surface are dependent on properties such as topography, surface charges, and chemistry [30, 31].

The porous three-dimensional scaffold acts as a temporary ECM for the physical support of cells, their adhesion, growth, and differentiation [32–34], and the adequate sizing of pores is essential in scaffold design for tissue engineering, providing sufficient space for cell migration, adhesion, proliferation, and the ingrowth of new bone tissue [35, 36]. The advantage of porous materials is their ability to provide biological anchorage for the surrounding bony tissue via ingrowth of mineralized tissue into the pore space [37]. A porous-surfaced implant could improve early implant stability and resistance of mechanical removal [38]. The high porosity (65–70%) and the broad pores (diameter of 350 to 550 *μ*m) should be sufficient to enable an ample nutrition supply inside the scaffold. The trabecular Ti scaffolds used in this work have an average porosity of 65% and a pore diameter of 640 *μ*m, which is currently being used clinically as a bone implant. The PLGA and PLGA/HA scaffolds have higher porosity than the Ti scaffolds, ranging between 75% and 84%, but smaller pore sizes, averaging 300 *μ*m; these parameters can affect cell growth and proliferation. If the pores are too small, cell migration is limited, resulting in the formation of a cellular capsule around the edges of the scaffold; this in turn can limit diffusion of nutrients resulting in necrotic regions within the construct. Conversely, if pores are too large there is a decrease in surface area limiting cell adhesion [9, 39]. By facilitating capillary formation, pores greater than 300 *μ*m lead to direct osteogenesis while pores smaller than 300 *μ*m can encourage osteochondral ossification [12, 24, 27].

In a previous study O'Brien et al. [40] showed that specific surface area decreases with increasing pore size; it is hypothesized that the effect of specific surface area is due to the ligand density available for integrin-binding after initial seeding [15]. Moreover PLGA scaffold pore size has been selected as a function of scaffold mechanical properties: large pore size makes the scaffold more fragile and decrease surface density. Thus the scaffold pore size selected is a good compromise between their mechanical resistance and their biocompatible properties. This concept is not applicable to Titanium scaffolds because their mechanical properties are not sensibly related to scaffold pore size. For this material the pore size selected seems to be to most suitable to improve osteointegration [39].

Scaffolds with smaller pores have a greater surface area which provides increased sites for initial cellular attachment postseeding; scaffolds with the largest pores facilitate a higher rate of scaffold infiltration with even cell distribution. Cells migrate into the centre of the scaffold resulting in the absence of cell aggregation; this demonstrates that cell migration increases with increasing pore size [41]. It is important to identify the upper limits in pore sizes as large pores may compromise the mechanical properties of the scaffolds by increasing void volume [12]. Therefore maintaining a balance between the optimal pore size for cell migration and specific surface area for cell attachment is essential [16].

The mechanical properties of the two materials are very different. As reported in the literature, the mechanical properties of a scaffold should resemble, the closest as possible those of bones.

Elastic modulus of cortical bone (long bones) ranges between 17 and 20 GPa for longitudinal axis, 6 and 13 GPa for transversal axis; elastic modulus of spongious bone ranges between 50–100 MPa [42]. Looking at these references parameters, the values of elastic modulus for Titanium are more close to those of cortical bone; this makes Titanium a good material for repair of long bones fractures. The evaluated polymer scaffolds have values of elastic modulus lower than those of both bones and titanium. Nevertheless, addition of HA to PLGA greatly improves the mechanical properties of polymeric scaffolds, above all as tensile properties.

The hASCs isolated from the subcutaneous adipose tissue of the hip contained a distinct cell population which expressed the stem cell markers CD73, CD90, and CD105; these results are consistent with others [17–20].

Morphological investigation with SEM demonstrated that hASCs grown in osteogenic medium for 28 days produced an abundant and homogeneous extracellular matrix (Figures 4, 5, and 6) containing proteins such as alkaline phosphatase and type I collagen (Table 3) extracted from the scaffold/cells construct. The amount of alkaline phosphatase, an extracellular protein necessary for matrix mineralization [35, 36], extracted from the Ti scaffold/cells construct was twofold higher on Ti scaffolds with respect to PLGA and PLGA/HA scaffolds. Deposition of type I collagen that represents 90% of the bone matrix and osteocalcin, an extracellular protein necessary for matrix mineralization, was highly decreased on PLGA and PLGA/HA scaffolds if compared to Ti scaffolds. Differently to Ti samples, collagen reduction was much higher on PLGA (5-fold) with respect to PLGA/HA (3-fold) scaffolds whereas for osteocalcin was 3 fold on PLGA and 2-fold on PLGA/HA. These results are quite interesting showing an order in the biomaterials predilection for cells adhesion and proliferation: Ti > PLGA/HA > PLGA.

Bone type-I collagen, designated [alfa1(I)2alfa2], comprises 85–90% of the total organic bone matrix, and its synthesis is upregulated at the proliferation stage and downregulated during the subsequent stages [43–45]. The deposition of a larger amount of type-1 collagen and osteocalcin on Ti scaffold in comparison to PLGA and PLGA/HA scaffolds may suggest that the type of scaffold could favour osteoblast proliferation and differentiation and promote bone ECM deposition.

Although no reports have evaluated which scaffold is optimal for ASC culture and differentiation, we used a PLGA scaffold because of its stability and utility for bone tissue engineering by surface modification.

The extracellular matrix calcification was confirmed with von Kossa staining after 28 days of differentiation in monolayer and by X-ray microanalysis on scaffolds.

The results achieved demonstrate that PLGA and PLGA/HA are biocompatible and that scaffolds made of these polymers are suitable for cell proliferation; the higher amount of protein matrix on Ti scaffold with respect to the PLGA and PLGA/HA scaffolds leads to conclude that not only the type of material but the structure significantly affects cell proliferation. The structural parameters for scaffold to be used in bone repair application resulted to be those shown by Trabecular Titanium scaffold. Since addition of HA to polymer improves the scaffold mechanical properties keeping their biocompatibility, the composite scaffolds should be further investigated.

Despite the fact that material science technology has resulted in clear improvements in the field of bone substitution medicine, no adequate bone substitute has been developed.

A new generation of scaffolds is needed with appropriate porosity, degradation rates, and mechanical properties. New processing techniques, namely, those that allow for the development of scaffolds with improved mechanical properties without influencing the porosity and interconnectivity should be studied and developed [46].

## Figures and Tables

**Table 1:** Apparent density, porosity, and pore size of PLGA and PLGA/HAP scaffolds.

Scaffold type	Apparent density (g/L)^a^	Porosity (%)^a^	Pore size (*μ*m)^b^
PLGA	0.12 ± 0.03	83.77 ± 0.62	200–350
PLGA/HA	0.19 ± 0.01	75.44 ± 0.4	200–300

^a^Determined by Displacement method (solvent - Ethanol); ^b^Determined by SEM.

**Table 2:** Compression and tensile properties of PLGA and PLGA/HA scaffolds.

Compression test				Tensile test		
Scaffold type	Ec1 (Mpa)	Ec2 (Mpa)	Ec3 (Mpa)	Et (Mpa)	Ts (Mpa)	UTS (Mpa)
PLGA	1.66 ± 0.76	0.88 ± 0.17	3.07 ± 0.28	2.88 ± 1.44	0.13±0.03	0.15 ± 0.03
PLGA/HA	4.76 ± 2.32	1.07 ± 0.16	—	15.68 ± 4.41	0.17 ± 0.04	0.35 ± 0.12

**Table 3:** Normalized amount of the extracellular matrix proteins secreted and deposited throughout PLGA, PLGA/HA and Ti scaffolds cultured in osteogenic medium for 28 days.

Type of scaffold	Alkaline phosphatase	Type I collagen (pg/cell per scaffold)	Osteocalcin
PLGA	2.60 ± 0.05	5.18 ± 0.1	2.00 ± 0.04
PLGA/HA	2.91 ± 0.01	8.80 ± 0.2	3.00 ± 0.2
Trabecular Ti	4.00*± 0.13	26.50*± 0.15	6.00 ± 0.07

*P ≤ .05 versus PLGA and PLGA/HA scaffolds.
